# Scanning the evidence: A pilot study evaluating the accuracy of sonographer reporting in the Republic of Ireland

**DOI:** 10.1177/1742271X261425842

**Published:** 2026-03-19

**Authors:** Jacqueline Tyler, Lorelei Waring, Gareth C Bolton, Paul K Miller

**Affiliations:** 1 University of Cumbria, Lancaster, UK

**Keywords:** Sonographer reporting, ultrasound report, Ireland

## Abstract

**Introduction:**

In the Republic of Ireland (ROI), sonographers undertake ultrasound examinations but are limited to providing provisional reports requiring radiologist verification, except in obstetrics. ROI sonographers are educated to the same postgraduate standard as their independently reporting UK counterparts. This study investigates the comparability of sonographer and radiologist reporting throughout ROI.

**Methods:**

This clinical audit included sonographers from six ROI hospital groups. Each submitted 400 randomly selected non-obstetric examinations over 6 months. Provisional sonographer reports were reviewed by supporting radiologists and allocated agreement scores based on the Riley 2010 grading system.

**Results:**

In total, 6037 examinations were audited of which 99.6% achieved acceptable grades (Grades 1 and 2), 0.35% (21) were Grade 3, and only two requiring upgraded pathology classification. No reports were Grade 4 (unacceptable).

**Conclusion:**

This large, multicentre audit demonstrates the accuracy of sonographer reporting in the ROI with an agreement score of over 99%. Given this evidence and the substantial international literature demonstrating that sonographer reporting is both safe and effective, health authorities, regulatory agencies and imaging departments should consider developing systems to allow sonographers to report independently without routine radiologist oversight. This could release radiologists to undertake other duties, reducing waiting lists in both ultrasound and broader radiology.

## Introduction

With the advancement of Ultrasound technology, there is an increased demand for diagnostic ultrasound services worldwide. Ultrasound imaging is the second most performed diagnostic test in the United Kingdom, with over 800,000 examinations performed between January 2022 and January 2023 alone.^1^ With increasing demand, many countries have moved away from the traditional practice of physician-performed ultrasound to utilising other health professionals to undertake the scan.^2^ However, the responsibility for reporting the ultrasound scans varies significantly worldwide. Some countries require a physician to administer a report based upon the saved scan images; others allow the healthcare professionals to provide a provisional report which requires verification from a physician.^3^

The provision of ultrasound services within the UK lies primarily with the sonographer; these services are well-established and essential to keeping up with demand.^3^ The UK system allows a sonographer to provide an independent, evaluative report of the scan, enabling scans to be performed and reported without the need for physician input. While the model is highly successful, it is also unique. Its achievement relies partly on rigorous postgraduate education, with many NHS sonographers completing CASE-accredited courses, though the title itself remains unregulated.

In 2017, the British Medical Ultrasound Society (BMUS) provided a position statement asserting that, due to the skills required to acquire, interpret and report the scan, the final report should be provided by the individual undertaking the scan itself. This is supported by clinically based evidence from the United Kingdom, which has demonstrated a successful model of independent sonographer reporting for over 30 years.^3^

At time of writing, there remains a paucity of research addressing the specific benefits that independent sonographer reporting has generated within radiology. Although evidence is limited, there is a reduction in service cost by utilising advanced practice radiographers compared to radiologists, who also benefit by contributing to their education.^4^ Research has demonstrated that when a specialist sonographer was trained in Head and Neck examinations, it not only improved patient wait times but also created cost savings of approximately £60,000 per year, with the additional benefit of releasing radiologists.^5^ This allowed them to perform other tasks, thereby addressing waiting lists elsewhere in the radiology department.

There was an 8.4% annual increase in the number of non-obstetric ultrasound scans (NOUS) between 2007/2008 and 2014/2015.^2^ From 2010 to 2015, England saw an increase in the consultant radiologist’s workforce of 16%; these figures do not represent whole-time equivalence.^2^ The waiting period for patients during this time has only increased by 1%. Given that during this period the increase in demand for CT and MRI scans has also increased by 29% and 26% respectively, the limited increase in wait time for Ultrasound examinations must be accredited to Sonographers, without whom the gap in the radiologist workforce vs the increasing demand would have had a devastating impact on patient care.

While Ireland is one of the countries that utilises speciality radiographers (sonographers) to undertake the ultrasound examination, outside of Obstetrics, they are limited to providing a provisional report which a Radiologist will verify. Sonographer education in Ireland is often provided by CASE-accredited courses such as those provided at University College Dublin or by UK institutions. The educational level of Irish sonographers is equivocal to that of those within the United Kingdom who report independently.

According to a study by Harrison et al.,^6^ the major barriers to independent sonographer reporting throughout Europe are as follows:

- Lack of appropriate education provision.
- Lack of professional registration.
- Resistance from radiologists/ other medical doctors.
- Legislation.

Lack of professional registration has not prevented the expansion of radiographers and other professionals into sonography. Since the introduction of a graduate-entry MSc Ultrasound programme in the United Kingdom, new sonographers have been employed by NHS trusts despite not having a regulated professional body (they currently voluntarily enrol with the Register for Clinical Technologists – RCT). Many sonographers in Ireland come from a radiography background and therefore can apply for professional registration via this route.

A study by Pedersen et al.^7^ demonstrated that it was felt across Europe that there was resistance from radiologists to allow independent reporting by sonographers because of ‘a certain amount of protectionism’. Gibbs, Edwards and Harrison further noted that these attitudes were experienced within the United Kingdom, but the fears were assuaged by the publication of many studies co-authored by sonographers and radiologists.^3^ European studies have documented radiologist resistance to independent sonographer reporting, attributed to protectionism, though UK experience demonstrates these concerns can be assuaged through collaborative research. Addressing this resistance requires focusing on tangible benefits: radiologists would gain time for other clinical duties by no longer reviewing sonographer reports. This is particularly relevant in Ireland, where, despite a 26% increase since 2011, radiologist numbers remain below OECD averages.

The legal responsibility for the ultrasound report falls with the author and as such this can cause some anxiety among radiographers due to the vulnerability and fear of legal action being taken.^3^ These fears are somewhat assuaged in the United Kingdom by clear, professional guidelines provided by the representative professional bodies and ultrasound societies. The Royal College of Radiologists recognises the significant role-played by sonographers in the United Kingdom within their guidelines by delegating certain duties and responsibilities.^3^ Ireland and the United Kingdom also currently require that sonographers have professional indemnity insurance which would act as a level of protection should legal action arise which will provide a level of reassurance for the reporting professional.

## Methods

The aim of this study is to determine if Irish sonographer reporting is equivalent to that of the radiologist. To assess this, a clinical audit methodology is utilised, this is a commonly employed tool in healthcare, to assess care quality. It confirms alignment with current standards and guidelines, connecting to clinical governance and effectiveness to identify best practice evidence.^8^ Within the field of medical ultrasound, the standard audit methodology utilised involves the review of stored ultrasound images alongside a review of the associated ultrasound reports.^9^

Ethical approval was not needed for this study as Clinical Audit fall out of scope of the Health Service Executive (HSE) Research Ethics Committee.

This clinical audit evaluates existing practice against established standards to inform local service improvement,^10^ distinguishing it from hypothesis-driven research seeking generalisable knowledge. To ensure the pilot study accurately represented the entire demographic and reflected practices across ROI, the HSE National Director of Acute Operations contacted all hospital group Chief executive officer (CEOs). They were invited to submit criteria-based applications to participate in the six-month pilot project from March to September 2023.

Applicants were required to submit their curriculum vitae and a site declaration form stating the support of the Radiography Services Manager, Lead Consultant Radiologist, Supporting Ultrasound Radiologist and CEO/General Manager, alongside a commitment to financially support the pilot from the Hospital CEO/Finance Director. Eligibility criteria can be seen in Table 1.

**Table 1.** Demonstrating pilot eligibility criteria.

Eligibility Criteria
1. Declaration of support form signed by Radiography Services Manager, Lead Consultant Radiologist, Supporting Ultrasound Radiologist and CEO/General Manager
2. Commitment to financially support the pilot by the Hospital CEO/Finance Director – successful candidate to be paid at RSM 1 level for the duration of the pilot.
3. QQI Level 9 or equivalent in ultrasound (copy of qualification to be submitted).
4. Minimum of 3 years clinical experience post ultrasound qualification.
5. Minimum of 30 weeks per annum working in ultrasound in this 3-year period.
6. Applicants must be currently completing full provisional reports on the PACS/RIS system.
7. If sites have multiple Sonographers expressing interest in participating – the RSM/Lead Radiologist must select one sonographer to represent their site.
8. Applicant must be able to commit to the pilot for a 6-month period and must submit 400 examinations in total– 200 in 1st 3 months and 200 in 2nd 3-month period.

All applications were reviewed and assessed by the Advanced Practice Radiography Working Group. Hospital site selection was primarily based on the individual sonographer’s qualifications and experience, and only public hospital applications were accepted. The original intention was to include approximately ten sites, however due to significant interest, the Advanced Practice Radiography Working Group extended the pilot to include 16 participants across 16 hospital sites (see Table 2). One hospital withdrew from the project late in the process.

**Table 2.** Demonstrating pilot participants from each region.

Hospital group	Participating hospitals
South/Southwest	Cork University Hospital
	University Hospital Waterford
	University Hospital Kerry
Ireland East	Mater Misericordiae University Hospital
	St. Vincent's University Hospital
Dublin Midlands	St. James’ Hospital
	Tallaght University Hospital
	Midland Regional Hospital Portlaoise
Saolta Hospital Group	Letterkenny University Hospital
	Mayo Primary Care Health Centres
	Sligo General Hospital
	Portiuncula University Hospital
Royal College of Surgeons in Ireland	Connolly Hospital
Children’s Health Ireland	Temple Street
	Crumlin
University of Limerick	N/a

All participants included in the pilot study were pseudo-anonymised by the working group and allocated a number between 1 and 15. Patients were scanned by the participating sonographer, who provided a provisional report. The images and provisional reports for the examinations were reviewed by the supporting radiologist, an agreement score allocated and recorded on the audit form. All participants were required to submit the anonymised images and both reports in intervals throughout the study via NIMIS study share.

It is common practice to assign generic agreement scores when auditing sonographer reports and images, as this presents a measurable standard and can be applied across all fields. The audit undertaken by Riley et al.^10^ represents an early example of this audit method. This agreement grading system was adopted for the audit as it considered the potential for disagreements which may alter patient care during score allocation. It produces quantifiable results from qualitative analysis to facilitate data analysis and evaluation. The categories are also not over-complex, which is beneficial for such a large study involving so many auditors. Details of the agreement score are highlighted in Table 3.

**Table 3.** Description of the agreement scores adapted from Riley et al.^10^

Agreement score	Description of the agreement score
Grade 1	Agree completely with the sonographer’s report
Grade 2	Minor discrepancy unlikely to alter patient care
Grade 3	Potentially significant discrepancy
Grade 4	Definite, significant discrepancy likely to have adverse consequences for patient

Following their results, Riley, Groves and Chandramohan proposed that a minimum agreement score across Grades 1 and 2 of 95% should be the standard for future quality assurance audit.^10^

The Advanced Practice Radiography Working Group reviewed the data collection periodically to ensure the participants were working within the criteria. All cases allocated with an agreement score of 3 or 4 were independently reviewed by the Working Group as well as a sample of Grade 1 and 2 cases.

## Results

On completion of the pilot study, a total of 6037 cases had been audited across the 15 participants. All cases included in this study were non-obstetric ultrasound (NOUS) examinations. Table 4 presents the range of agreement scores for each participant. Table 5 demonstrates the number of reports in each grade category.

**Table 4.** Overall agreement range for each participant represented as the total number in each category.

	1	2	3	4	5	6	7	8	9	10	11	12	13	14	15
G1	382	402	402	380	390	364	353	347	348	379	399	394	394	400	358
G2	18	6	6	20	22	35	37	50	49	19	10	6	6	0	40
G3	0	0	0	0	0	1	10	3	3	2	0	0	0	0	2
G4	0	0	0	0	0	0	0	0	0	0	0	0	0	0	0

**Table 5.** Total number of reports in each grade category.

	Total number in each category across all participants	Percentage of total across all participants
Grade 1	5692	94.28
Grade 2	324	5.40
Grade 3	21	0.35
Grade 4	0	0.00

A high level of agreement was recorded between the auditee and the auditors. The data shows that a total of 99.6% of audited examinations fell into Grades 1 (t = 5692) and 2 (t = 324), with the remaining cases falling into category 3 (t = 21). No Category 4 disagreements were recorded.

Of the total audited examinations, 21 (including one double examination) were allocated a Grade 3, accounting for 0.35%. In most instances, the radiologist concurred with the examination findings; however, they disagreed with the proposed additional imaging or recommended follow-up. Notably, a significant number of the Grade 3 cases related to disagreement in the interpretation and management of thyroid nodules. However, these Grade 3 numbers are still low and sit well within the acceptable disagreement standard.

A total of 324 Grade 2 disagreements were recorded, representing 5.4% of the total audited cases. On review of a selection of Grade 2 cases, it was noted that the disagreements related to specific wording of the sonographer report and suggested follow-up, but importantly did not alter patient care.

In two of the cases reviewed, there was no discernible disagreement between the provisional report and the final report; it is suspected that this may be related to a data input error.

A total of 5692 Grade 1 agreement scores were recorded, representing 94.28% of the total audited cases. On review of a selection of Grade 1 cases, the auditing radiologist agreed with the findings on the scan, the report, and any suggested follow-up. However, in one case, the radiologist changed the wording of the report to offer additional actionable advice.

## Discussion

To fully understand the value of this audit, it is pertinent to consider the results in conjunction with previous studies. Key to this is an understanding that while agreement scoring ultimately measures concordance between sonographer and radiologist interpretations, it serves only as a proxy for diagnostic accuracy, rather than definitive validation against patient outcomes. The performance of sonographers versus radiologists in reporting ultrasound examinations has been audited and published from as early as the mid-1990s. Bates et al.^11^ undertook one of the earliest audits of sonographer reporting of NOUS in the United Kingdom, auditing a total of 1046 examinations, which yielded an acceptable agreement rate of 99.3% between the reporting sonographer and the radiologist. In 94% of cases, the sonographer's report was deemed to present an accurate record of the findings and in 6.3% of cases, the radiologist added additional minor comments to the report.

Leslie et al.^12^ conducted a study where 100 patients were scanned consecutively by a sonographer and a consultant radiologist using the same equipment, with each report blinded to the other’s findings. Disagreements led to a third scan by an independent consultant radiologist. This study showed 96% of examinations met acceptable standards, with 93% in complete agreement and 3% minor disagreement. Although effective, these ‘double scanning’ audits are time-consuming and unsuitable for large-scale or ongoing audits, thus the study only includes 100 examinations by a single sonographer and radiologist.

Freeman et al.^9^ published the results of one of the most extensive UK audits of sonographer reporting. This was undertaken over an 11-year period between 2010 and 2020 utilising the Trusts internal audit model and the external audit tool introduced by BMUS in 2011. For the internal audit the images and reports from a total of 3731 randomly selected NOUS examinations were retrospectively reviewed by 6 consultant radiologists and one consultant sonographer. The internal audit system devised allocated an agreement score to each examination of Grades 1–5, setting an acceptable agreement standard of 95% of examination within Grades 3–5 as per Riley et al.^10^ Categories 1 and 2 were deemed to represent unacceptable report quality:

- Grade 5 – Agree with report.
- Grade 4 – General agreement but minor additional comment required.
- Grade 3 – Additional differential diagnosis on review of images.
- Grade 2 – Significant disagreement with interpretation of images.
- Grade 1 – Clinical question not answered and cannot be inferred from report.

The agreement standard varied across the 11-year period, ranging from 92.5% to 99.2%; however, the acceptable standard was exceeded for the final six years of the audit. Across the 11-year period, the mean agreement score was 95.5%. This pilot audit shares several similarities with the Freeman et al.^9^ study, as both included data from a significant pool of sonographers. It also recruited a larger cohort of auditors than the Riley et al.^10^ study. However, the Freeman et al.^9^ study is limited to one hospital trust, located in the Northeast of England, and does not represent the wider picture across the United Kingdom.

Although the differences in methodology employed by these studies may present challenges when comparatively evaluating these data, the reviewed literature indicates that suitably trained sonographers can provide accurate and effective ultrasound reports. When we consider the results reported above, we can see that standards of sonographer reporting within the ROI are commensurate with the wider ‘profession’. Indeed, the percentage of examinations deemed to be within the acceptable standard Grades 1 and 2 (99.6%) exceeds the other documented studies. This level of accuracy has significant implications for Irish healthcare workforce planning. Given that radiologists in the ROI currently verify every sonographer report outside obstetrics, the findings suggest this verification step could be safely eliminated.

There remains relatively limited evidence of independent sonographer reporting outside of the United Kingdom, although ultrasound is undertaken by sonographers in many countries,^3^ so it is useful to consider the international picture.

In the United States, the role of a sonographer is complex, and the training and education routes are varied. Sonographers generally provide a provisional report to the interpreting radiologist, often having their reports checked and verified prior to the patient leaving the departments.^13^ There is limited evidence of published audit data within the literature; however, a study undertaken in 2006 explored the provisional reporting accuracy of two sonographers over a 12-month period acceptable and the authors reported that the results exceeded expectations, demonstrating 99.7% Grade A and B agreements for participant 1 and a 99.8% for participant 2.^14^

High levels of agreement between preliminary reports issued by sonographers and final radiologists’ reports are also evident in audits undertaken in Australia, Canada and Norway.^15–17^ Although the first sonographers were trained in Norway in 2008, and they do practice and produce full interpretive reports, their introduction has faced resistance from radiologists. To support the wider inclusion of sonographer reporting into the workforce Hofmann and Vikestad^16^ undertook an audit looking at the accuracy of 5 sonographers reporting practices. 244 patients were scanned by the sonographers and one of four advanced radiologists. The reports were not allocated an agreement score, but the results demonstrated that the sonographer and radiologist came to the same conclusion in 94.49% of the examinations.

The educational routes and imaging standards in Canada and Australia are comparable to the United Kingdom and ROI and like the ROI there is a push to integrate independent reporting into to the role of the sonographer in these countries.^3^ Dima et al.^15^ recognised that more research was needed to provide evidence of Canadian sonographer’s capabilities when it comes to reporting ultrasound examinations. This retrospective audit of 429 reports over one month employed the Riley grading system, achieving 95% acceptable agreement,^10^ and allocated the same agreement score of 1 to 4 to each examination. The overall agreement score fell in the acceptable range with 95% of cases in the Grades 1 and 2 categories.^15^

Several studies evaluating the accuracy of Australian sonographers reporting accuracy have been published including a small-scale study undertaken by Schneider et al,^17^ which compared 86 provisional sonographer reports to the final radiologist reports. A high accuracy rate was recorded with 98.9% of the examinations falling within the acceptable categories 1 and 2, however, this was a small-scale study and only included upper abdominal examinations.

A more extensive audit was undertaken by Williams et al,^18^ which reviewed a total of 532 examinations undertaken by eight sonographers over a 3-month period. The sonographer preliminary report was compared to the final radiologist's report, and an agreement score was allocated. In this audit, all examinations fell within categories 1 (97.2%) and 2 (2.8%). This study included a significant sample size and included the full range of ultrasound examinations undertaken by the sonographers.

A particularly valuable contribution to this evidence base comes from three large studies, examining normal examinations, abnormal findings, and obstetric examinations, conducted in New Zealand.^19–21^ These employed a different methodological approach to those detailed above, correlating sonographer-radiologist discrepancies with final clinical outcomes rather than simply measuring agreement. In sum, the outcomes-based analysis revealed that when disagreement occurred between sonographer and radiologist reports, approximately half of the discrepancies were attributable to radiologist error. This finding provides compelling evidence that independent sonographer reporting is not only safe, but that mandatory radiologist verification may not enhance, and could potentially compromise, report accuracy.

While these studies do not consider the reporting practices or the accuracy of sonographer reporting across the full sonographer demographic, they do represent a cross section of the sonography workforce in the United Kingdom, the United States, Europe (Norway), Canada and Australia. It is recognised that the audit methodology does vary across these studies; however, they demonstrate that there is very little difference in reporting accuracy and sonographer capabilities in the interpretation of ultrasound images when compared to a radiologist, regardless of country of origin and reporting responsibilities. For the ROI specifically, where radiologist numbers remain below OECD averages despite recent workforce growth, independent sonographer reporting represents a potential practical mechanism to address capacity constraints without compromising diagnostic quality.

The subjectivity and operator dependence of US imaging must be considered when assessing any audit data.^22^ There is notable variation within the data gathered for this pilot study regarding what the different radiologists involved deemed to be Grade 2 and Grade 3 disagreements. This is to be expected, as review of static images is an opinion-based interpretation and what radiologists may term a significant disagreement will not always be equivocal. It is particularly evident in this study due to the scale of the project and the involvement of multiple radiology departments covering the whole of the ROI. This research project appears to be unique as no other audit studies evaluating at the reporting accuracy of sonographers across a whole nation have been found.

Many of the Grade 3 image interpretation disagreements in this study related to imaging of the thyroid gland and specifically the grading of thyroid nodules. In all the cases reviewed, the sonographers graded the nodules higher than the radiologist, suggesting that they are more cautious in their reporting. However, without follow-up data, uncertainty remains about whose interpretation was correct. This is the same for all Grade 3 disagreements related to a difference in interpretation of the ultrasound appearances. Interestingly, in the audit published by Leslie et al,^12^ it was noted that of the seven disagreements on the findings of the scan, the sonographer was correct in three cases and the radiologist in four. A number of studies, meanwhile, found that in cases of disagreement, it was the sonographer (rather than the radiologist) who was correct.^9,19,20^ This finding is further supported by important recent research. Alfuraih et al.^23^ demonstrated that sonographers in Saudi Arabia outperformed radiologists in TI-RADS classification of thyroid nodules. Conversely, Necas et al.^21^ found that radiologist review of obstetric reports in New Zealand introduced discrepancies and de-novo errors, effectively reducing report quality compared to independent sonographer reporting.

Interpretation errors are inherent to medical imaging evaluation, with ultrasound particularly subject to these.^24^ Hofmann and Vikestad^16^ noted that in 3.3% of cases where the radiologists agreed with the sonographers’ findings, further investigation revealed both to be incorrect in their interpretation. So, while clinical audit of sonographer practices is an essential and valuable part of a diagnostic ultrasound service, it should be remembered that review of static images is still an opinion-based retrospective interpretation and is not always correct.^22^ The difference between an actual error in interpretation and a difference of opinion in interpretation is an arbitrary division. It is important to note, however, that the number of Grade 3 disagreements is small, and these nuances would not have a significant impact on the final figures or statistics.

### Strengths and weaknesses of the study

This audit evaluates the full remit of sonographer NOUS practices in ROI, offering a clear picture across all specialities and applications of ultrasound. No published literature could be found looking at the reporting standards of sonographers over such a wide geographical area; other reviewed studies are limited to a single hospital trust or ultrasound service. This pilot study offers a valuable insight into the standards of reporting in the ROI and allows for a comparison of these standards across all regions, demonstrating that there is little variation in the standards across the country. In addition, the high number of participants and examinations included in the audit increase the impact of the results and accuracy of the findings, as large volume audits produce significantly higher quality data.^9^

A well-established audit methodology was utilised for this audit, although review of static ultrasound images has inherent limitations,^22^ it is a widely accepted method of obtaining information on sonographer performance which can be undertaken retrospectively on a regular basis to drive quality improvements or service development.^9^

In terms of weaknesses, first, despite the rich data set, the involvement of 15 different auditors increases the potential for differing opinions. No formal inter-auditor calibration was undertaken prior to the audit; however, the low disagreement rates suggest broad consensus in interpretation standards across sites. However, when considering the number of disagreements overall in the data, it is unlikely this would have any impact on overall statistics. Second, sonographer reports were audited by consultant radiologists from the same radiology department, meaning there may be some bias due to the existing relationships, although it is in the interest of all parties to adopt an impartial approach to the audit. External audit can minimise this limitation; however, it is challenging to facilitate, considering the size and timescale of the study.^9^

Third, the opinion of the radiologist was assumed to be correct with no arbitration; however, as highlighted in key studies, this is not always necessarily the case.^9,12^ Logistically, incorporating a process of arbitration would be challenging in view of the size and geographical spread of the audit. All Grade 3 disagreements were reviewed by the oversight group, meaning any significant trends could have been highlighted and addressed if required. Future work incorporating outcome-based validation, as demonstrated in the New Zealand cohort studies cited, would strengthen claims regarding diagnostic accuracy beyond inter-rater agreement.^19–21^

Fourth, the examinations chosen for inclusion were selected by the sonographer and not randomly selected by a third party which may have introduced some unconscious bias. While sonographers were asked to include consecutive eligible cases, it is acknowledged that the absence of independent verification of case selection may have influenced the high agreement rates observed. Finally, as this was a review of still images, other factors relevant to image interpretation such as patient factors were not considered when assigning an agreement score.

## Conclusion

The results of this pilot study highlight that sonographers in the ROI have the capabilities to independently report their own ultrasound examinations, potentially benefitting the sonographer workforce as well as the wider radiology community. The findings demonstrate an agreement rate of 99.6% between sonographer and radiologist reports, substantially exceeding the established acceptable standard, and matching or surpassing comparable international audits. This evidence, combined with the documented body of global research, all demonstrating consistently high accuracy rates in sonographer reporting, provides a compelling case for change in this domain.

Given the increasingly strong evidence that sonographer reporting is both safe and effective, it is appropriate to recommend that health authorities, regulatory agencies and imaging departments in the ROI develop systems and processes to allow sonographers to report independently without the routine oversight of radiologists. Such a transition would not only acknowledge the demonstrated competence of appropriately trained sonographers but could also bring tangible benefits to the wider healthcare system. Removal of the compulsory report verification process by radiologists could improve the patient journey via reduced waiting times, while concurrently releasing radiologists to undertake other duties, with the corollary impact of addressing waiting lists in other areas of radiology where specialist input is essential.

While determining quality and setting standards remains essential in this highly operator-dependent modality, there is a strong argument that the evidence base now supports a move towards independent sonographer reporting in the ROI, mirroring the successful model established in the United Kingdom over the past three decades. Implementation should be staged, beginning with defined examination categories and credentialed practitioners, supported by robust governance structures including mandatory checking of said credentials, regular revalidation, and ongoing audit cycles to ensure safety and consistency. This study provides valuable evidence not only for the ROI but for the wider international ultrasound community, many of whom are similarly evaluating such changes to address workforce challenges while maintaining high standards of patient care.
